# A Response-Time-Based Latent Response Mixture Model for Identifying and Modeling Careless and Insufficient Effort Responding in Survey Data

**DOI:** 10.1007/s11336-021-09817-7

**Published:** 2021-12-02

**Authors:** Esther Ulitzsch, Steffi Pohl, Lale Khorramdel, Ulf Kroehne, Matthias von Davier

**Affiliations:** 1 IPN–Leibniz Institute for Science and Mathematics Education, Olshausenstraße 62, 24118 Kiel, Germany; 2 Freie Universität Berlin, Berlin, Germany; 3 Boston College, Chestnut Hill, USA; 4 DIPF–Leibniz Institute for Research and Information in Education, Frankfurt, Germany

**Keywords:** careless responses, data screening, response times, item response theory, mixture modeling

## Abstract

Careless and insufficient effort responding (C/IER) can pose a major threat to data quality and, as such, to validity of inferences drawn from questionnaire data. A rich body of methods aiming at its detection has been developed. Most of these methods can detect only specific types of C/IER patterns. However, typically different types of C/IER patterns occur within one data set and need to be accounted for. We present a model-based approach for detecting manifold manifestations of C/IER at once. This is achieved by leveraging response time (RT) information available from computer-administered questionnaires and integrating theoretical considerations on C/IER with recent psychometric modeling approaches. The approach a) takes the specifics of attentive response behavior on questionnaires into account by incorporating the distance–difficulty hypothesis, b) allows for attentiveness to vary on the screen-by-respondent level, c) allows for respondents with different trait and speed levels to differ in their attentiveness, and d) at once deals with various response patterns arising from C/IER. The approach makes use of item-level RTs. An adapted version for aggregated RTs is presented that supports screening for C/IER behavior on the respondent level. Parameter recovery is investigated in a simulation study. The approach is illustrated in an empirical example, comparing different RT measures and contrasting the proposed model-based procedure against indicator-based multiple-hurdle approaches.

**Supplementary Information:**

The online version supplementary material available at 10.1007/s11336-021-09817-7.

## Introduction

**Fig. 1:**
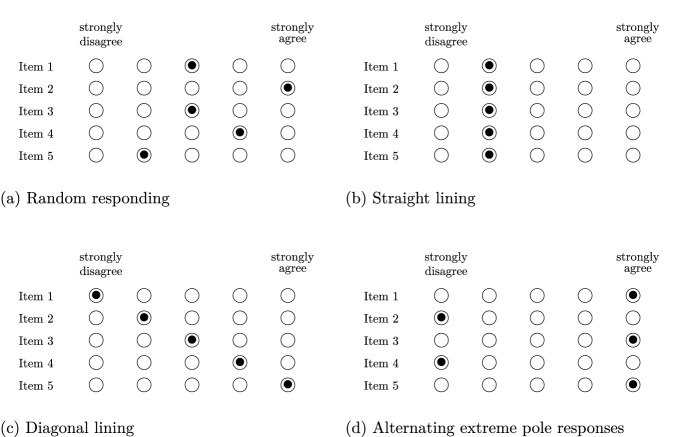
Schematic illustration of different careless and insufficient effort response patterns

Research in psychology, educational and social sciences heavily relies on questionnaire data.^1^ Careless and insufficient effort responding (C/IER), referring to a “survey response set in which a person responds to items without sufficient regard to the content of the items and/or survey instructions” (Huang, Liu, & Bowling, 2015, p. 828), may pose a major threat to data quality, and, as such, to validity of inferences drawn from questionnaire data. C/IE respondents are assumed to quickly proceed through the survey, and instead of providing high-quality data by attentively evaluating the item, retrieving relevant information, and selecting a relevant response, to choose response options that do not reflect the trait to be measured. Careless responses, although not reflecting respondents’ trait levels, may not necessarily be random (as in Fig. 1a), but might follow distinct patterns Curran & Denison, 2019; DeSimone, DeSimone, Harms, & Wood, 2018; Kroehne, Buchholz, & Goldhammer, April 2019; Meade & Craig, 2012) such as straight lining (see Fig. 1b), diagonal lining (see Fig. 1c), or alternating extreme pole responses (see Fig. 1d).

When left unconsidered, C/IER can have detrimental effects on conclusions drawn from questionnaire data. These range from introducing systematic variance to—depending on dominant C/IER patterns—both attenuated or inflated associations among constructs of interests (Huang et al., 2015; McGrath, Mitchell, Kim, & Hough, 2010), and distorted psychometric properties such as reliability and factor structure (DeSimone et al., 2018; Huang, Curran, Keeney, Poposki, & DeShon, 2012; Schmitt & Stuits, 1985; Woods, 2006).

Conceptually, C/IER can be understood as a special case of response style behavior. Response styles refer to a systematic response tendency irrespective of the item content (Baumgartner & Steenkamp, 2001). Vast literature exists proposing sophisticated model-based solutions for identifying and modeling response styles (see Böckenholt & Meiser, 2017; Khorramdel, Jeon, & Leigh Wang, 2019, for overviews over current solutions). Usually, in approaches for identifying and modeling response styles, observed responses are allowed to be affected by both the respondents’ content trait and their response styles. Under C/IER, in contrast, responses may not be reflective of the respondents’ trait levels whatsoever. What is more, response style approaches have commonly been tailored to detecting and modeling specific types of response styles, such as mid point, extreme, or acquiescent response styles. Nevertheless, recent approaches allow for modeling and detecting multiple types of response styles simultaneously (Adams, Bolt, Deng, Smith, & Baker, 2019; Bolt, Lu, & Kim, 2014; Takagishi, van de Velden, & Yadohisa, 2019). Similar to these approaches, researchers may not have presumptions on the specific type of C/IER in their data due to its various possible manifestations.

Previous approaches that specifically aim at detecting C/IER usually support the detection of some types of C/IER behavior, but are insensitive to others (see Curran, 2016; Niessen, Meijer, & Tendeiro, 2016, for overviews and comparisons). In this article, we present a model-based approach for detecting manifold manifestations of C/IER at once. This is achieved by leveraging response time (RT) information available from computer-administered questionnaires and integrating theoretical considerations on C/IER with recent psychometric modeling approaches. We specifically make use of psychometric models that have been developed in the context of low effort on cognitive assessments and adapt them to the case of C/IER in non-cognitive assessments.

In the following, we first briefly review previous procedures for C/IER detection. We then delineate how drawing on recent method developments for detecting low effort on cognitive assessments can assist overcoming some of the limitations of these previous procedures, and present a model-based approach that leverages RTs for detecting and modeling multiple types of C/IER. When questionnaire items are administered as item batteries with multiple questions on one screen, timing data is oftentimes recorded at the screen-level. If further log data such as time-stamped log events are available, item-level RTs can be reconstructed (see Kroehne et al., April 2019; Kroehne & Goldhammer, 2018). To equip researchers with tools for both types of timing data, we first introduce the model-based approach considering RTs on the item level. We then provide an adapted version for RTs aggregated on the screen-level. Parameter recovery is investigated in a simulation study. We illustrate the approach using data from the Programme for International Student Assessment 2015 (PISA, OECD, 2017) background questionnaire and compare and contrast it against previous indicator-based procedures to C/IER.

## Previous Approaches for Detecting and Dealing with Careless and Insufficient Effort Responding

### Attention Check/Bogus/Instructional Manipulation Check Items

Oftentimes, researchers administer attention check, instructional manipulation check, or bogus items for drawing inferences on the attentiveness of respondents. These are items that researchers presume attentive respondent will answer in the same way (e.g., disagreement with “All my friends are aliens”; Curran, 2016; Meade & Craig, 2012). A response other than the expected one is taken as an indicator of C/IER. Such items, however, have to be used with precaution since extensive use might confuse attentive respondents (Meade & Craig, 2012). In addition, Curran and Hauser (2019) found that some respondents do not provide the expected answer to those items even after reading them aloud, suggesting that (attentively) reading the item does not inevitably lead to choosing the intended response.

### Response Pattern Analyses

Since C/IER is assumed to result in respondents choosing response options by mechanisms other than according to their trait levels, C/IER should come with response patterns that differ from attentive response patterns. Response–pattern-based approaches to C/IER have been designed for detecting different possible patterns arising from C/IER and involve a) assessing invariability of response patterns, b) assessing individual consistency in responses, c) outlier detection, or d) person-fit statistics. Only recently, machine learning methods have been suggested, which, however, require access to an adequate training data set. We will shortly review response–pattern-based methods based on a prominent example for each. Exhaustive overviews and discussions of other response–pattern-based indicators are given in Curran (2016), Meade and Craig (2012), and Niessen et al. (2016).

#### Response Invariability

The assumption underlying response invariability indicators as, for instance, derived from long string analyses is that C/IE respondents straight line. The long string index is constructed by examining the longest sequence of subsequently occurring identical responses for each respondent (Johnson, 2005). In order to disentangle extreme trait levels from straight lining, this approach requires differently pooled items or to mix items that refer to different traits.

#### Response Consistency

Response consistency indicators are built on the rationale that attentive response patterns are internally consistent, while C/IE response patterns are dominated by random responses (Curran, 2016; Jackson, 1976). A fairly simple measure of response consistency is the even–odd index, given by the within-person correlation between the responses to odd-numbered and even-numbered items belonging to the same scale. When multiple scales are administered, these correlations are averaged across scales. Large values indicate higher within-person consistency of response patterns and, as such, presumably lower levels of C/IER (Curran, 2016; Huang et al., 2012).

#### Outlier Analysis

Since the majority of responses is likely to stem from attentive response processes (see Hauser & Schwarz, 2016; Thomas & Clifford, 2017, for comparisons of different samples), C/IE responses can also be seen as outliers that deviate from typical response patterns. Mahalanobis distance (Mahalanobis, 1936) provides a measure of the multivariate distance between the respondent’s response vector and the vector of sample means (Ehlers, Greene-Shortridge, Weekley, & Zajack, 2009; Maniaci & Rogge, 2014; Meade & Craig, 2012). However, Mahalanobis distance can be influenced by too much normality in C/IE responses (arising when respondents randomly choose categories around the mid point, Curran, 2016) and thus performs well for detecting uniformly distributed but fails detecting normally distributed random responses (Meade & Craig, 2012).

#### Person-Fit Statistics

Person-fit statistics can be used to identify response patterns that are unlikely to be observed given an assumed statistical model for item responses. A common person-fit statistic employed for C/IER is the number of Guttman errors in item response theory (IRT) models. In the case of dichotomously scored responses, the number of Guttmann errors is given by the number of item pairs ordered by difficulty with a 0 on the item that is easier to endorse and a 1 on the item that is more difficult to endorse (Meijer, 1994). An extension to ordered polytomous data can be found in Emons (2008). In a similar vein, the $l^p_z$ statistic has been employed (developed by Drasgow, Levine, & Williams, 1985; employed for C/IER by Niessen et al., 2016). This statistic quantifies the likelihood of observing a response vector under a given IRT model. In the case that the $l^p_z$ statistic is very low, the response pattern strongly deviates from what could be expected based on the employed IRT model, and the pattern is classified as inconsistent. In the context of C/IER, person-fit statistics have predominantly been evaluated for identifying uniform random responses (Niessen et al., 2016).

#### Combining Multiple Response–Pattern-Based Indicators

A major limitation of response–pattern-based methods for detecting C/IER is that different measures have been designed for detecting different types of C/IER. For instance, long string analysis has been designed to detect C/IER in terms of straight lining, it is, however, insensitive to other forms of C/IER such as random responding or diagonal lining. Conversely, consistency indicators are insensitive to straight lining since this results in consistency of response patterns (Curran, 2016). Accordingly, when applied to both empirical and simulated data, different methods may show positive, negative, or no agreement (Meade & Craig, 2012; Niessen et al., 2016). Within one survey, different types of C/IER are likely to be present (Meade & Craig, 2012) and need to be dealt with.

Due to the different performance of C/IER measures under different C/IE response patterns, it is commonly recommended to draw conclusions on C/IER based on multiple measures (Curran, 2016; Meade & Craig, 2012; Niessen et al., 2016). Curran (2016) suggested a multiple-hurdle approach that filters out respondents with the most extreme values on each indicator considered based on conservative cut-off values and provided guidelines on how to decide on these cut-off values. Curran (2016), however, also noted that any cut-off setting will to some degree falsely classify attentive as C/IE respondents and/or vice versa.^2^ Since in a multiple-hurdle approach multiple cut-off values need to be set, setting cut-offs too high for some indicators and too low for others may result in a complex interplay of different misclassifications that is yet not well understood. Further, depending on the indicators employed, the multiple-hurdle approach may also be affected by the order in which the indicators are considered. This is due to the fact that some indicators, such as Mahalanobis distance or person-fit statistics, are affected by which respondents have been filtered out in preceding hurdles.

As an alternative, multiple measures can be aggregated. Huang et al. (2015), for instance, performed principal component analysis on multiple measures for C/IER and subsequently employed the first factor extracted as a measure of C/IER. Since the first factor extracted from principal component analysis might only capture the dominant C/IE response patterns, this procedure may not control for all types of C/IER.

Note that, again, approaches that combine information from different indicators only support the detection of C/IER patterns to which the employed indicators are sensitive to. As such, these approaches considerably alleviate, but not entirely eradicate the issue of focusing on specific patterns of C/IER behavior.

#### Employing Supervised Machine Learning

To avoid making assumptions concerning the specific types of C/IER patterns—or attentive response patterns, for that matter—Schmidt, and Gnambs (2020) suggested to employ supervised machine learning techniques, with the algorithm being trained on a data set for which it is known which respondents displayed attentive and C/IER behavior, e.g., on a data set stemming from an experiment manipulating instructions on how to approach the questionnaire. Note that the training and test data sets need to be based on the same questionnaire. The approach is limited in that it requires access to an adequate training data set and is based on the assumption that both attentive and C/IE responses follow a structure that is comparable to the respective structures in the training data. This assumption may be violated when respondents do not comply with instructions in the study for obtaining the training data or when respondents being instructed to show C/IER behavior do not behave in a comparable manner to those displaying C/IER behavior in out-of-lab conditions.

### Response Time Analyses

Due to the absence of cognitive processing required for attentively evaluating the item, retrieving relevant information, and selecting a relevant response, short RTs spent on single items can be seen as indicators of C/IER. Since in computer-administered questionnaires, multiple items are oftentimes displayed on one screen, item-level RTs might not always be at hand and time spent on screen or the survey as a whole may be used as an aggregated proxy (Huang et al., 2012). Previous research utilizing RTs has focused on time spent on screen and on the whole survey, classifying respondents with screen or completion times below a pre-defined threshold as showing C/IER. The thresholds are commonly defined either based on an educated guess on the minimum amount of time required for an attentive response (Huang et al., 2012; Meade & Craig, 2012) or are created using visual inspection of the RT distribution (Kroehne et al., April 2019; Wise, 2017).

One of the major advantages of RT-based over response–pattern-based indicators is that these do not entail presumptions on the specific C/IER patterns. In support of this, Huang et al. (2012) and Huang et al. (2015) found high agreement between RT-based and various other, response–pattern-based indicators. Niessen et al. (2016) compared different methods using both empirical and simulated data. RT-based indicators outperformed response–pattern-based indicators in terms of sensitivity to different C/IER patterns. For validating different measures of C/IER, Meade and Craig (2012) performed factor mixture modeling analyses on facet-score level data and regressed class membership on different indicators of C/IER. Meade and Craig (2012) reported two classes: one class with high and one with low factor loadings, with the latter being interpreted as a response class with high prevalence of C/IER. Meade and Craig (2012) found that completion times could well predict latent class membership, that is, could well predict whether respondents generated facet scores showing high or low association with the construct to be measured.

Nevertheless, Meade and Craig (2012) argued against the use of RTs as single indicators of C/IER due to practical considerations concerning thresholds. While very short RTs or very few time spent on the survey can well be seen as indicators of C/IER, RTs above a set threshold may or may not stem from C/IER. That is, attentive and inattentive RT distributions are likely to overlap, potentially resulting in misclassifications by RT-based threshold methods (Curran, 2016; Meade & Craig, 2012). It has therefore been recommended to apply a sequential approach that classifies C/IER first, based on RTs and second, using response–pattern-based indicators for respondents with longer RTs (Maniaci & Rogge, 2014; Meade & Craig, 2012).

### Dealing with Careless and Insufficient Effort Responding

Previous approaches that deal with C/IER by excluding either responses or cases from the analyses may yield biased conclusions on item and structural parameters (i.e., variances, covariances, or regression coefficients of the traits to be measured), especially when the mechanisms underlying C/IER are not distinct from the traits to be measured (see Köhler, Pohl, & Carstensen, 2017; Pohl, Gräfe, & Rose, 2014; Rose, 2013; Rose, von Davier, & Xu, 2010, for related research on the treatment of nonignorable missing responses). Commonly, respondents with C/IER indicator values falling below a certain cut-off value are eliminated from further analyses. Removing presumable C/IE respondents from further analyses comes with the assumption that the missing values induced by this procedure are ignorable, implying that the constructs to be measured and the mechanisms underlying C/IER are unrelated. Empirical research, however, has found the extent to which C/IER behavior is shown to be related to person characteristics and common constructs of interest such as education (Kim, Dykema, Stevenson, Black, & Moberg, 2018) or personality (Bowling et al., 2016; Huang et al., 2015; Maniaci & Rogge, 2014), rendering this assumption likely to be violated. In this case, filtering can yield biased conclusions (Deribo, Kroehne, & Goldhammer, 2021; Ulitzsch, von Davier, & Pohl, 2020). In addition, C/IER may vary across the assessment and respondents who display C/IER on some parts of the assessment might still provide valid responses to others. Indeed, respondents are more likely to respond randomly toward the middle or end of long questionnaires (Baer, Ballenger, Berry, & Wetter, 1997; Berry et al., 1992), while probably providing valid responses at the beginning. Discarding all responses of respondents who have been identified to show C/IER at some point of the questionnaire thus also discards their valid responses.

## Approaches for Disengaged Responding Developed in the Context of Cognitive Assessments

The distinction between attentive response behavior and C/IER in non-cognitive assessments shows many parallels to the distinction between solution and disengaged rapid guessing behavior in cognitive assessment. In cognitive assessments, solution behavior is assumed to result in item responses reflecting “what the test taker knows and can do” (Wise, 2017, p. 52). Its counterpart, non-effortful test-taking behavior, is defined as “quickly proceeding through the test without applying [...] knowledge, skills, and abilities” (Wise & Gao, 2017, p. 384). Research on non-effortful test-taking behavior has predominantly focused on rapid guessing as one possible manifestation of non-effortful test-taking behavior. Hence, veins of research on C/IER on the one hand and rapid guessing behavior on the other hand both assume disengaged, respectively inattentive, responding to require less time for its execution than engaged responding (Kroehne et al., April 2019). Sophisticated models for detecting disengaged responding in cognitive assessments have been developed. These could be adapted to non-cognitive assessments and may thereby enhance identification of C/IER.

With the rise of computer-based assessment and the related availability of log data, a rapidly growing body of methods emerged aiming at identification of rapid guessing behavior in cognitive assessments. Primarily, these methods leverage RT data either by defining RT-based scoring rules, with responses associated with RTs below a pre-defined threshold being classified as rapid guesses (Goldhammer, Martens, Christoph, & Lüdtke, 2016; Guo et al., 2016; Lee & Jia, 2014; Wise, Kingsbury, Thomason, & Kong, April 2004; Wise & Ma, April 2012; Wise, Pastor, & Kong, 2009) or by utilizing RT information in mixture modeling approaches, explicating different data-generating processes for RTs and responses associated with solution and (rapid) guessing behavior (Nagy & Ulitzsch, 2021; Schnipke & Scrams, 1997; Ulitzsch et al., 2020; Wang & Xu, 2015).

A recent example for mixture modeling approaches is the speed-accuracy+engagement (SA+E) model developed by Ulitzsch et al. (2020). The SA+E model allows for rapid guessing behavior to vary at the item-by-person level. For the probability of observing a correct response under solution behavior, the SA+E model assumes an IRT model to hold. Probability correct for rapid guesses is assumed to correspond to the probability of guessing correct at chance level. RTs associated with solution behavior are modeled as a function of person speed and the item’s time intensity (see also van der Linden, 2007). RTs associated with rapid guessing are assumed not to depend on person or item characteristics and to be shorter than those associated with valid responses. Item-by-person mixing proportions are modeled with a latent response approach as a function of person engagement and item engagement difficulty employing an IRT model. By doing so, the model allows assessing how the tendency to show rapid guessing behavior relates to ability and speed as well as identifying items that are likely to evoke rapid guessing behavior. The SA+E model overcomes major limitations of previously developed approaches for the identification of rapid guessing behavior. First, as a purely model-based approach, the SA+E model does not require setting an RT threshold and allows for overlapping RT distributions, potentially resulting in fewer misclassifications when there is strong overlap of RT distributions associated with solution and rapid guessing behavior. Second, the model allows for rapid guessing behavior to vary across both items and persons and does, as such, not discard valid responses from test takers rapidly guessing only on some parts of the test. Third, since the tendency to show rapid guessing behavior is modeled jointly with ability and speed, the model does not rely on the assumption that ability and the mechanism underlying rapid guessing behavior are unrelated.

Given the parallels of C/IER and rapid guessing behavior as processes resulting in (possibly) fast responses not reflecting the traits to be measured, it deems promising to build on these recent developments in cognitive assessments to improve the identification of C/IER. Indeed, the literature on the identification of rapid guessing behavior has already stimulated research on the identification of C/IER. Huang et al. (2012), for instance, built their rationale for classifying respondents with completion times below a pre-defined threshold on methods for detecting rapid guessing behavior developed by Wise and DeMars (2006). Nevertheless, concepts and methods developed in the context of cognitive assessments are not directly applicable to the context of non-cognitive assessments. First, methods for identifying rapid guessing behavior have been developed in the context of dichotomously scored responses. Non-cognitive assessments, however, primarily rely on Likert scales for measuring constructs of interests, that is, most often entail (ordered) polytomous response data. Second, and more importantly, methods for non-effortful responding developed in the context of cognitive assessment are concerned with probability correct, the analysis of responses for detecting C/IER, however, is concerned with the chosen response option itself. Third, in non-cognitive assessment data, the relationship between RTs and the trait to be measured is likely to deviate from the linear relationship commonly assumed in models that integrate RT information with IRT models in the context of cognitive assessment. One example is the distance–difficulty hypothesis (Ferrando & Lorenzo-Seva, 2007; Kuncel & Fiske, 1974), assuming that responses take more time when an item is well targeted to the trait of a person, while responses can be given rather quickly when the item thresholds deviate strongly from the trait level of the person. This mimics the fact that statements can be faster endorsed (or not endorsed), when persons are sure of their response. While mixture models for identifying rapid guessing behavior in cognitive assessments are very promising, they need to be adapted to suit the specifics of response behavior in non-cognitive assessments.

## Proposed Approach

The presented approach for identifying and modeling C/IER behavior is a latent response model for computer-administered questionnaires in which item-level RTs are available. Building on Ulitzsch et al. (2020), the approach a) takes the specifics of attentive response behavior in non-cognitive assessments into account by incorporating the distance–difficulty hypothesis, b) allows for attentiveness to vary on the screen-by-respondent level, c) allows for respondents with different trait and speed levels to differ in their attentiveness, and d) can deal with various response patterns arising from C/IER. The approach assumes that respondents have a constant probability to provide either attentive or C/IE responses on all items on a screen and that respondents do not switch between response modes on a given screen. They can, however, switch from C/IE to attentive responding and vice versa between screens.^3^

In the presented approach, latent attentiveness indicators $\Delta _{is}$ denote whether respondent *i*, $i=1,\ldots ,N$, was attentive when approaching screen *s*, $s=1,\ldots S$, $(\Delta _{is}=1)$ or not $(\Delta _{is}=0)$. While the attentiveness status itself is not observable, it is assumed to be associated with different data-generating processes underlying responses and RTs. When approaching a screen attentively, respondents are assumed to generate responses according to their trait levels on all item administered on the screen. When showing C/IER, respondents are assumed to choose response options that do not reflect their trait level. C/IER behavior can have various manifestations, including choosing randomly, marking patterns, such as straight or diagonal lines, or alternating extreme pole responses.

For reason of simplicity, however, without loss of generality, we present the approach assuming the same number of response options for all items, and that all items measuring a trait are displayed on one screen, and that each screen contains items measuring one trait only. Concerning the relationship between RTs and trait levels, we focus on the distance–difficulty hypothesis as a special case.

### Attentive Behavior

#### Item Responses

When being attentive, respondents are assumed to respond to all items displayed on screen *s* according to their trait levels. Different IRT models such as the graded response model (Samejima, 2016) or the generalized partial credit model (Muraki, 1997) can be employed to model attentive responses. Here, we present the model with a generalized partial credit model for item responses $x_{ijs} \in \{0. \ldots , K\}$, containing person *i*’s response to the *j*th item, $j=1,\ldots ,J_s$, displayed on screen *s*, with *K* giving the highest possible response category for the considered items. That is, under $\Delta _{is} = 1$, we model the probability of respondent *i* to choose category *k*, $k=1,\ldots ,K$, on the *j*th item displayed on screen *s* as

1 $$\begin{aligned} p(x_{ijs} = k | \Delta _{is} = 1) = \frac{\exp \left( \sum _{l=0}^{k} v_{js}\eta _{is} - b_{jsl}\right) }{\sum _{r=0}^{K}\exp \left( \sum _{l=0}^{r} v_{js}\eta _{is} - b_{jsl}\right) }\qquad \text {with } \sum _{l=0}^{0} v_{js}\eta _{is} - b_{jsl}\equiv 0. \end{aligned}$$

Here, $\eta _{is}$ denotes respondents *i*’s level on the *s*th trait. The parameters $b_{jsl}$ and $v_{js}$ give the *l*th step difficulty and discrimination of item *j* measuring latent trait *s*, respectively.

#### Response Times

When associated with attentive responses, RTs $t_{ijs}$, denoting the time respondent *i* spent on the *j*th item displayed on screen *s*, are assumed to follow a lognormal distribution governed by the respondent’s speed $\tau _i$ and the item’s time intensity $\beta _{js}$ (see Ulitzsch et al., 2020; van der Linden, 2007). The distance–difficulty relationship between the respondents’ trait levels and their RTs is incorporated following Molenaar, Tuerlinckx, and van der Maas (2015) by regressing log RTs on the absolute weighted distance between the respondent’s trait level and the middle step difficulty parameter $o_{js}$. In the case of four response categories with the three step difficulty parameters $b_{js1}$, $b_{js2}$, and $b_{js3}$, for instance, $o_{js}$ is given by $b_{js2}$.^4^ That is, attentive RTs are modeled as

2 $$\begin{aligned} \ln \left( t_{ijs}| \Delta _{is} = 1\right) \sim \mathcal {N}\left( \beta _{js} - \tau _{i} - \gamma |v_{js}\eta _{is} - o_{js}| , \sigma ^2_{A}\right) , \end{aligned}$$

with $\gamma$ denoting the distance–difficulty parameter. Note that different approaches exist for incorporating the distance–difficulty relationship between traits and RTs (see Ranger, 2013, for an overview). Further, the relationship of the distance between the respondent’s trait level and the middle step difficulty parameter and RTs must not necessarily be linear but may take other functional forms.

We assume a common residual variance $\sigma ^2_{A}$ (see van der Linden, 2007). Note that a common speed factor is assumed across all measured traits, that is, it is assumed that respondents approach all screens to which they respond attentively with the same speed level.

### Careless and Insufficient Effort Behavior

#### Item Responses

Category probabilities that are not reflective of person or item characteristics are estimated for inattentive responses, that is,

3 $$\begin{aligned} p(x_{ijs} = k |\Delta _{is} = 0) = \kappa _k\qquad \text {with } \sum _{k=0}^{K} \kappa _k = 1. \end{aligned}$$

Note that $\kappa _k$ gives the marginal probability over all types of C/IER patterns of inattentively choosing category *k*. Hence, the model is capable of capturing various types of C/IER patterns that all result in no relationship with the measured trait. The model does, however, not allow disentangling groups of respondents with different C/IER patterns.

#### Response Times

In line with mixture modeling approaches for rapid guessing behavior in cognitive assessments (Schnipke & Scrams, 1997; Ulitzsch et al., 2020; Wang & Xu, 2015), we assume RTs associated with C/IE responses to be unaffected by person or item characteristics. Hence, for RTs associated with C/IER, we assume a lognormal distribution governed by a common mean $\beta _{C}$ and a common variance $\sigma _C^2$:

4 $$\begin{aligned} \ln \left( t_{ijs}| \Delta _{is} = 0\right) \sim \mathcal {N}\left( \beta _{C}, \sigma _C^2\right) . \end{aligned}$$

It is further assumed that C/IER requires less time than evaluating the item, retrieving relevant information, and selecting a relevant response. This mirrors the assumption of rapid, disengaged guesses in cognitive assessment to be shorter than responses stemming from good faith attempts to solve an item (Wise, 2017). Hence, following Ulitzsch et al. (2020), time intensities for attentive RTs $\beta _{js}$ are defined as the sum of the C/IER mean $\beta _{C}$ and an item-specific, positive offset parameter $\beta ^{*}_{js}$. That is,

5 $$\begin{aligned} \beta _{js} = \beta _{C} + \beta ^{*}_{js} \qquad \text {where } \beta ^{*}_{js} \ge 0. \end{aligned}$$

The offset parameter $\beta ^{*}_{js}$ indicates how much longer respondents commonly require to generate an attentive response to the *j*the item presented on screen *s* rather than showing C/IER. Note that RT distributions associated with attentive and careless responses are allowed to overlap, such that also responses associated with longer RTs may be classified as C/IER.

### Higher-Order Structures

The attentiveness status $\Delta _{is}$ of respondent *i* on screen *s* is not observable. It, however, determines the measurement properties of the observed responses and associated RTs and thus represents a latent response variable (see Maris, 1995). In line with Ulitzsch et al. (2020), latent response variables $\Delta _{is}$ are modeled using a Rasch model as a function of the respondent’s attentiveness $\psi _i$ and the screen’s attentiveness difficulty $\iota _s$, that is

6 $$\begin{aligned} p\left( \Delta _{is}=1\right) =\frac{\exp \left( \psi _i-\iota _s\right) }{1+\exp \left( \psi _i-\iota _s\right) }. \end{aligned}$$

This supports investigating respondent characteristics associated with low attentiveness and allows identifying screens evoking C/IER behavior. For instance, if screens administered at the end of the survey are more likely to evoke C/IER behavior, they can be expected to have higher attentiveness difficulties.

Person parameters are assumed to be multivariate normally distributed with mean vector and covariance matrix

7 $$\begin{aligned} \varvec{\mu }=\left( \mu _{\psi }, \mu _{\tau }, \mu _{\eta _{1}}, \dots , \mu _{\eta _{S}}\right) \qquad \text {and} \qquad \varvec{\Sigma }=\begin{pmatrix} \sigma ^2_{\psi } &{} \sigma _{\psi \tau } &{} \sigma _{\psi \eta _1} &{} \dots &{} \sigma _{\psi \eta _S} \\ \sigma _{\psi \tau } &{} \sigma ^2_{\tau } &{} \sigma _{\tau \eta _1} &{} \dots &{} \sigma _{\tau \eta _S} \\ \sigma _{\psi \eta _1} &{} \sigma _{\tau \eta _1} &{} \sigma ^2_{\eta _1} &{} \dots &{} \sigma _{\eta _1\eta _S} \\ \vdots &{} \vdots &{} \ddots &{} \vdots &{} \vdots \\ \sigma _{\psi \eta _S} &{} \sigma _{\tau \eta _S} &{} \sigma _{\eta _1\eta _S} &{} \dots &{}\sigma ^2_{\eta _S} \\ \end{pmatrix}. \end{aligned}$$

For identifying the model, we set person parameter means to zero. When a generalized partial credit model is employed for attentive responses, the model can be identified by setting trait variances to one. Item parameters are modeled as fixed effects. The proposed model’s likelihood can be written as

8 $$\begin{aligned} \mathcal {L}= & {} \prod _{i=1}^N \prod _{s=1}^S \left( p\left( \Delta _{is}=1|\psi _i,\iota _s\right) \prod _{j=1}^{J_s} p\left( x_{ijs}|\eta _{is}, v_{js}, \varvec{b}_{js}\right) ^{\left( 1-d^{(x)}_{ijs}\right) }f\left( t_{ijs}|\tau _i, \eta _{is}, \beta _{js}, \gamma , v_{js}, o_{js}, \sigma ^2_A\right) ^{\left( 1-d^{(t)}_{ijs}\right) }\right. \nonumber \\&\left. +\left( 1-p\left( \Delta _{is}=1|\psi _i,\iota _s\right) \right) \prod _{j=1}^{J_s} p\left( x_{ijs}|\varvec{\kappa }\right) ^{\left( 1-d^{(x)}_{ijs}\right) }f\left( t_{ijs}|\beta _{C}, \sigma ^2_C\right) ^{\left( 1-d^{(t)}_{ijs}\right) }\right) h\left( \varvec{\psi },\varvec{\tau },\varvec{\eta }_1, \dots , \varvec{\eta }_S | \varvec{\mu }, \varvec{\Sigma }\right) ,\nonumber \\ \end{aligned}$$

The first and second component represent the component models for attentive and C/IER behavior, respectively. Here, *N*, *S*, and $J_s$ denote the number of respondents, screens (and, as such, traits to be measured), and number of items administered on screen *s*. The term $h(\varvec{\tau },\varvec{\eta }_1, \dots , \varvec{\eta }_S | \varvec{\mu }, \varvec{\Sigma })$ denotes the multivariate normal density of the person parameters. The terms for responses and RTs incorporate the assumption of independence of response and RT indicators given the second-order variables of the model. The indicators $d^{(x)}_{ijs}$ and $d^{(t)}_{ijs}$ denote whether or not a response or RT of respondent *i* to the *j*th item measuring trait *s* is available, with $d^{(x)}_{ijs}=0$ denoting an observed and $d^{(x)}_{ijs}=1$ a missing response and $d^{(t)}_{ijs}=0$ denoting an observed and $d^{(t)}_{ijs}=1$ a missing RT.^5^

### Model Modification for Screen-Level Timing Data

In computer-administered questionnaires, item-level RTs are not always available as these oftentimes require additional, sophisticated data processing (Kroehne et al., April 2019; Kroehne & Goldhammer, 2018). In most surveys (e.g., in PISA, OECD, 2017), only timing data on the screen-level (i.e. aggregated RTs) are available in public use files. To make the approach applicable for a broad audience, we present a model modification for more readily available screen-level timing data. The adapted model is a simplified version of the model for item-level RTs and assumes that respondents approach the assessment either with no or full attentiveness and that, as such, there are no response vectors in which both C/IE and attentive responses occur. Instead of employing a latent response approach, the adapted model assumes a respondent-specific attentiveness probability that is constant across screens and distinct from the traits to be measured, that is $p(\Delta _{is}=1) = \pi _i$. As such, as previous approaches for C/IER behavior, the model is well suited for scanning for C/IER behavior on the respondent level.

We make use of mean time spent on the items presented on screen *s*, defined as the total screen-level time divided by the number of items and denoted with $\bar{t}_{is}$, as a proxy for item-level RTs, and adapt the measurement model for aggregated RTs associated with attentive responses. Mean time spent on the items presented on screen *s* associated with inattentive responses is modeled according to Eq. 4, assuming a common mean and variance parameter. To adapt the measurement model for attentive aggregated RTs, we consider a screen- rather than an item-specific time intensity parameter $\beta _{s}$, determining the average time respondents require for providing attentive responses to the items presented on screen *s*.^6^ For considering the distance–difficulty relationship between the respondents’ trait levels and their RTs, we average the discrimination and middle step difficulty parameters of the items presented on screen *s*, taking the screen-level geometric mean of discriminations $v_{\varvec{\cdot } s}$ and the arithmetic mean of middle step difficulties $o_{\varvec{\cdot } s}$. The parameter $\gamma$ thus gives the average distance–difficulty effect for all items presented on a screen. Hence, the average time respondent *i* spent on the items presented on screen *s* is modeled as

9 $$\begin{aligned} \ln \left( \bar{t}_{is}|\Delta _{is}=1\right) \sim \mathcal {N}\left( \beta _{s} - \tau _{i} - \gamma |v_{\varvec{\cdot } s}\eta _{is} - o_{\varvec{\cdot } s}| , \sigma ^2_{A}\right) . \end{aligned}$$

Screen-level time intensity parameters are subject to the constraint

10 $$\begin{aligned} \beta _{s} = \beta _{C} + \beta ^{*}_{s}\qquad \text {where } \beta ^{*}_{s} \ge 0. \end{aligned}$$

For simplicity, attentiveness parameters are dropped from the multivariate normal distribution of person parameters. In the adapted model, the mean vector and covariance matrix of person parameters are given by

11 $$\begin{aligned} \varvec{\mu }=\left( \mu _{\tau }, \mu _{\eta _{1}}, \dots , \mu _{\eta _{S}}\right) \qquad \text {and} \qquad \varvec{\Sigma }=\begin{pmatrix} \sigma ^2_{\tau } &{} \sigma _{\tau \eta _1} &{} \dots &{} \sigma _{\tau \eta _S} \\ \sigma _{\tau \eta _1} &{} \sigma ^2_{\eta _1} &{} \dots &{} \sigma _{\eta _1\eta _S} \\ \vdots &{} \vdots &{} \ddots &{} \vdots \\ \sigma _{\tau \eta _S} &{} \sigma _{\eta _1\eta _S} &{} \dots &{}\sigma ^2_{\eta _S} \\ \end{pmatrix}. \end{aligned}$$

This yields the following likelihood for the adapted model

12 $$\begin{aligned} \mathcal {L}= & {} \prod _{i=1}^N \left( \pi _{i} \prod _{s=1}^S f\left( \bar{t}_{is}|\tau _i, \eta _{is}, \beta _{s}, \gamma , v_{\varvec{\cdot } s}, o_{\varvec{\cdot } s}, \sigma ^2_A\right) ^{\left( 1-d^{(t)}_{is}\right) } \prod _{j=1}^{J_s} p\left( x_{ijs}|\eta _{is}, v_{js}, \varvec{b}_{js}\right) ^{\left( 1-d^{(x)}_{ijs}\right) }\right. \nonumber \\&\left. +\,(1-\pi _{i}) \prod _{s=1}^S f\left( \bar{t}_{is}|\beta _{C}, \sigma ^2_C\right) ^{\left( 1-d^{(t)}_{is}\right) } \prod _{j=1}^{J_s} p(x_{ijs}|\varvec{\kappa })^{(1-d^{(x)}_{ijs})}\right) h\left( \varvec{\tau },\varvec{\eta }_1, \dots , \varvec{\eta }_S | \varvec{\mu }, \varvec{\Sigma }\right) .\nonumber \\ \end{aligned}$$

### Prior Distributions

For model estimation, we employ Bayesian estimation techniques. Priors are set in accordance with Ulitzsch et al. (2020). We employ an LKJ prior (Lewandowski, Kurowicka, & Joe, 2009) with shape 1 for the correlation matrix of person parameters $\varvec{\Omega }$, implying a uniform prior distribution for the correlation parameters. Half-Cauchy priors with location 0 and scale 5 are employed for all standard deviations, that is, the standard deviation of attentiveness $\sigma _\psi$ and speed $\sigma _\tau$, the residual standard deviation of log attentive RTs $\sigma _{A}$, and the common standard deviation of inattentive RTs $\sigma _{C}$, as well as item discriminations $v_{js}$. Diffuse normal priors with mean 0 and standard deviation 10 are employed for each step difficulty $b_{jsl}$, time intensity offset parameter $\beta ^*_{js}$, respectively $\beta ^*_{s}$, the distance–difficulty parameter $\gamma$ as well as the common mean $\beta _C$. For C/IER category probabilities, we suggest a diffuse Dirichlet prior with $\varvec{\kappa } \sim \text {Dir}(\varvec{1})$. For attentiveness probabilities $\pi _i$ in the model with screen-level timing data, we employ a Dirichlet prior, parameterized as $(\pi _i, 1-\pi _i) \sim \text {Dir}(\lambda \pi _{\mathcal {P}}, \lambda (1-\pi _{\mathcal {P}}))$, where $\pi _{\mathcal {P}}$ gives the population-level proportion of attentive respondents and $\lambda$ is a concentration parameter (see Kemp, Perfors, & Tenenbaum, 2007; Salakhutdinov, Tenenbaum, & Torralba, 2012). Population-level proportions of attentive and C/IE respondents are equipped with a diffuse Dirichlet prior, with $(\pi _{\mathcal {P}}, 1-\pi _{\mathcal {P}}) \sim \text {Dir}(1,1)$. The concentration parameter $\lambda$ is equipped with a half-Cauchy prior with location 0 and scale 5.

## Parameter Recovery

For investigating parameter recovery under realistic conditions, we generated data according to the model for item-level RTs. Data-generating values were chosen to resemble parameter estimates reported in the empirical example below. We considered a scenario with different C/IER patterns—uniform random responding, random responding around the endpoints, straight lining, and diagonal lining (see also Curran & Denison, 2019). This allows illustrating that the proposed approach indeed can deal with various patterns arising from C/IER, as long as C/IE responses do not reflect the trait to be measured and, on average, are not slower than attentive responses. We further investigated the potential loss in accuracy resulting from model simplification and aggregating RT information.

Under the investigated conditions, the data-generating model with item-level RTs yielded good parameter recovery and could deal well with the different simulated C/IER patterns as well as low C/IER rates. The model with screen-level timing data could well recover person parameter variances and correlations, step difficulties, marginal C/IER category probabilities as well as the distance–difficulty parameter. However, population-level C/IER rates were overestimated (median estimated C/IER rate: 9.39%, true C/IER rate: 5%). The misclassification of attentive as C/IE responses when using aggregated RTs was also mirrored in biased estimates of parameters related to the RT measurement model. Further, the loss of information on item-level RT variability resulted in estimates of RT residual variances close to zero. Detailed results of the simulation study are given in the supplementary material.

## Empirical Example

The empirical example serves a) to illustrate the insights that can be gained on the basis of the presented approach, b) to investigate differences between different measures of aggregated RTs available in large-scale assessment data as well as c) to compare the proposed approach to customary indicator-based procedures.

We took responses, screen-level timing data, and raw log data from the background questionnaire from PISA 2015 (PISA OECD, 2017). The PISA 2015 assessment focused on science as the major domain. For illustrating the proposed approach, we focused on the constructs “environmental awareness” and “enjoyment of science”, measured with 7 and 5 four-point Likert scale items, respectively. Items for either scale were presented on a single screen. For measuring environmental awareness, respondents were asked to gauge how informed they are on different environmental issues, e.g. nuclear waste or water shortage. Enjoyment of science was measured by asking respondents to express their level of agreement with statements such as “I generally have fun when I am learning science topics”. We analyzed data from the German sample, comprising $N=2847$ respondents. All analyses were performed using R version 3.6.3 (R Development Core Team, 2017).

### Implementation of Model-Based Approaches

To investigate differences in conclusions based on different RT measures, we conducted four separate analyses. We considered three measures for aggregated RTs, each aggregating different information of the response process, and one measure for item-level RTs. We used the LogFSM package (Kroehne, 2019) to extract item-level RTs from raw log events. The package implements the finite state machine (FSM) framework for log data presented by Kroehne and Goldhammer (2018). In the FSM framework, the RT for an item is defined as the difference between the time stamp associated with choosing a response option on that item and the time stamp associated with providing the preceding response. Note that in this framework the RT for the first item cannot be reconstructed as it is confounded with the time taken for reading the question stem. The FSM framework does not require items to be answered in a linear order (see Kroehne et al., April 2019, for details).

We considered aggregated RT measures that pose proxies for item-level RTs. As the most coarse proxy for item-level RTs, we considered the total time spent on screen (denoted with TT) divided by the number of items presented on the screen $J_s$. TT is oftentimes publicly available in computer-administered questionnaires. It, however, poses an aggregate of the time required for both reading and evaluating the question stem and the time required for reading, evaluating, and generating responses to the items presented on the screen. To separate these aspects, Kroehne et al. (April 2019) suggested to subtract the time elapsed until the first response (FRT) from TT. Note that FRT contains the time required for reading the question stem and answering the first item (see Kroehne & Goldhammer, 2018) . Hence, to eliminate reading time from the aggregated RT measure, we also considered TT−FRT divided by $J_s-1$ (denoted with TTFRT). Note that TT as given in the PISA 2015 data set is already cleansed. Since aggregated RT data provided in public use files are often cleansed, we aimed at investigating whether this preliminary data cleansing impacts conclusions and compared TTFRT against the average of reconstructed item-level RTs as a further measure for the average answering time (denoted with AAT). If the data cleansing procedure performed on aggregated RT data available in the PISA public use file does not impact conclusions, results for TTFRT and AAT should be similar. Note that both TTFRT and AAT are measures containing information on the average amount of time respondents required to generate responses to all but the first item answered.

Bayesian estimation was conducted using Stan version 2.19 (Carpenter et al., 2017) employing the rstan package version 2.19.3 (Guo, Gabry, & Goodrich, 2018). Stan code for both model types is provided in Appendix. For all models, we ran four Markov chain Monte Carlo (MCMC) chains with 4,000 iterations each, with the first half being employed as warm-up. The sampling procedure was assessed on the basis of potential scale reduction factor (PSRF) values, with PSRF values below 1.10 for all parameters being considered as satisfactory (Gelman & Rubin, 1992; Gelman & Shirley, 2011). In our first analyses, the model with item-level RTs did not converge. We therefore trimmed RTs, removing item-level RTs exceeding the 99.9th percentile of 90 seconds. Given the median of 2.51 seconds and the middle 50% range of [1.61; 3.79], RTs above 90 seconds are aberrantly large and occurred very rarely. In total, this led to the exclusion of 29 item-level RTs (i.e., 0.08% of the RT data points). We further excluded 13 AAT and 20 TTFRT values exceeding 90 seconds. No TT values were excluded as these did not show such aberrances. Note that none of the respondents exceeded 90 seconds on all available timing measures, such that all analyses were based on the same set of respondents. With this setup, for all models, the chains mixed well and no PSRF values below 1.10 were encountered.

### Implementation of Indicator-based Procedures

We compared and contrasted the proposed approach with the performance of customary indicator-based procedures. For fair comparisons with the presented approach, that can deal with different forms of C/IER, we implemented a multiple-hurdle approach (Curran, 2016). This approach uses multiple indicators that are sensitive to different aspects of C/IER. We focused on three commonly used indicators. Following Meade and Craig (2012), we first filtered respondents with extremely low RTs. For doing so, we employed AAT. Next, response vectors were scanned for C/IER employing the long string index. Finally, to balance off the long string index’s insensitivity to C/IER deviating from straight lining, Mahalanobis distance was employed to search the remaining response vectors for C/IER. In the multiple-hurdle approach, thresholds have to be set for each of its components. There are no globally applicable values for these thresholds, as the distributions of the indicators for careless and attentive respondents are scale-specific (Curran, 2016), depending, for instance, on the similarity of the administered items in the case of the long string index or the degree of normality in attentive and careless response distributions in the case of Mahalanobis distance. In order to evaluate the range of possible results and the impact of threshold settings, we implemented two sets of thresholds, choosing either a liberal or a conservative cut-off for all three indicators employed. Under the conservative threshold settings, mean time spent per item below 1 second was set to indicate C/IER. This value corresponds to the halved “educated guess” of 2 seconds for the time required for generating an attentive response by Huang et al. (2012), and is thus aimed at filtering out only the very extreme cases. We required the long string index to correspond to the total number of investigated items (i.e., 13) to be seen as indicating C/IER, which is the most conservative approach possible. Recall that squared Mahalanobis distance can be approximated by a $\chi ^2$ distribution with degrees of freedom corresponding to the number of variables (Rousseeuw & Van Zomeren, 1990). Respondents with squared Mahalanobis distances exceeding the 99th quantile of the $\chi ^2$ distribution with 13 degrees of freedom were classified as multivariate outliers, indicating C/IER. Under the liberal threshold settings, for the RT threshold, we employed the original “educated guess” by Huang et al. (2012), i.e., set the RT threshold to 2 seconds. For the long string index under liberal threshold settings, we classified respondents as careless when they chose the same response option on at least 5 out of 7 items on the environmental awareness scale and at least 4 out of 5 items on the enjoyment of science scale. Further, squared Mahalanobis distances exceeding the 95th quantile of the $\chi ^2$ distribution were seen as indicating C/IER. The long string index and Mahalanobis distance were calculated using the package careless (Yentes & Wilhelm, 2021). In contrasting the multiple-hurdle procedure against the proposed approach, we focused on differences in C/IER classifications.

### Results

#### Model-Based Approaches

Table 1 gives C/IER rates retrieved from all considered approaches. An overview over the remaining parameters from the model-based approaches with different RT measures is displayed in Table 2. By and large, differences between parameter estimates retrieved from models using aggregated and item-level RT information corroborated those observed in the simulation study. Further, TTFRT and AAT did not yield the same but comparable results, indicating that the data cleansing procedure performed on aggregated RT data available in the PISA public use file does not heavily impact conclusions.

**Table 1:** Rates of careless and insufficient effort responses of threshold-based multiple-hurdle and model-based approaches

Threshold-based		Model-based			
MH$_{\text {c}}$	MH$_{\text {l}}$	RT	TT	AAT	TTFRT
9.91%	22.83%	6.29%	8.28%	11.10%	13.02%

*Notes:* MH$_{\text {c}}$ and MH$_{\text {l}}$ denote the multiple-hurdle approach with conservative and liberal threshold settings, respectively; RT: item-level response times reconstructed from raw log events; TT: total time spent on screen divided by the number of items $J_s$; AAT: average item-level response time; TTFRT: difference between total time spent on screen and time to the first response divided by $J_s-1$.

We retrieved attentiveness difficulties of $\iota _1=-2.74$ and $\iota _2=-3.47$ for the environmental awareness and enjoyment of science screen, corresponding to screen-level C/IER rates of 7.97% and 3.93%, respectively. In the model with item-level RTs, in total 6.29% of responses were classified as C/IER. Implementing the proposed approach with different RT measures resulted in different conclusions concerning the prevalence of C/IER behavior; there was a twofold difference in C/IER rates between the measure yielding the lowest (item-level RTs) and highest (TTFRT) C/IER rate (see Table 1).

The models yielded rather different common mean and variance estimates for the distribution of C/IE RTs. While the model employing item-level RTs identified the common mean of inattentive RTs to be 0.74, corresponding to 2.10 seconds, TTFRT, for instance, yielded a much higher common mean of 1.11, corresponding to 3.00 seconds. While inattentive RTs as classified by the model employing item-level RTs strongly varied, the distribution of inattentive times in the AAT and TTFRT models showed very low variability. Note that the RT parameters for the model employing TT are not directly comparable with the other models as TT also contains information on reading time.

Consistent across RT measures, results suggest that, marginally, respondents tended to favor middle response categories. This is in line with cognitive theories on edge aversion in decision making processes when items do not need to be (or, as in the present case, are not) processed (Bar-Hillel, 2015).

Besides differences in the variability of speed, mirroring the variability of inattentive RTs, all models yielded comparable conclusions on the relationship between speed and the two traits. Both traits assessed were only weakly related to speed, indicating that respondents with different levels of environmental awareness and enjoyment of science did not considerably differ in the speed with which they generated attentive responses. Environmental awareness and enjoyment of science showed a medium positive correlation. The model with item-level RTs yielded small positive correlations of attentiveness with both traits, indicating that respondents with higher environmental awareness and enjoyment of science levels tended to approach the questionnaire with higher attentiveness. Such conclusions are not possible to draw from the models with aggregated RTs.

**Fig. 2:**
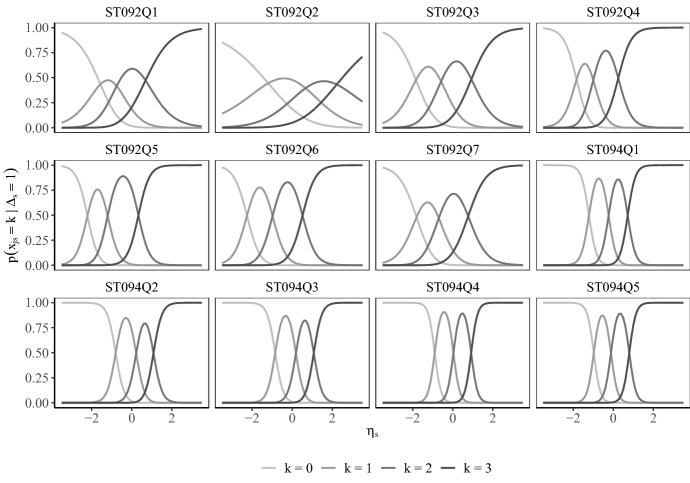
Category probabilities for attentive responses in the model with item-level response times. ST092 and ST094 denote items measuring environmental awareness and enjoyment of science

Parameters of the measurement model of attentive responses showed very high agreement, with correlations between parameters being above .95 between all models considered. Category probabilities are displayed in Fig. 2.

Time intensity offset parameters in the model with item-level RTs tended to decrease across the seven items of the environmental awareness screen (first two: $\beta ^*_{11}=0.46$ and $\beta ^*_{21}=0.68$; last two: $\beta ^*_{61}=0.13$ and $\beta ^*_{71}=0.00$), indicating that, on average, respondents increased their pace towards the end of this rather long screen. This was not the case for the five items of the enjoyment of science screen (first two: $\beta ^*_{12}=0.04$ and $\beta ^*_{22}=0.24$; last two: $\beta ^*_{42}=0.51$ and $\beta ^*_{52}=0.29$). For the model with TT, screen-specific time intensity offset parameters were 0.32 and 0.24. AAT and TTFRT yielded very low screen-specific time intensity offset parameters (0.05 and 0.00 for AAT and 0.11 and 0.02 for TTFRT), leading to the conclusion that, on average, log aggregated RTs associated with attentive and C/IER behavior in those models did not considerably differ.

With $\gamma =0.04$ in the model with item-level RTs, we found evidence for the distance–difficulty hypothesis in the selected two scales. That is, when the absolute difference between the respondent’s trait level and the item’s middle step difficulty increases by one standard deviation, attentive RTs are expected to decrease by the factor $\exp (-0.04)=0.96$. Comparable conclusions can be drawn on the basis of the models with aggregated RTs, with $\gamma$ ranging from 0.03 to 0.05.

**Table 2:** Results for different response time measures

	RT				TT				AAT				TTFRT			
	$\beta _C = 0.74, \sigma ^2_C= 0.78$				$\beta _C = 1.11, \sigma ^2_C= 0.45$				$\beta _C = 1.15, \sigma ^2_C= 0.03$				$\beta _C = 1.11, \sigma ^2_C= 0.05$			
Person parameter variances and correlations																
	$\psi$	$\tau$	$\eta _1$	$\eta _2$	$\tau$	$\eta _1$	$\eta _2$		$\tau$	$\eta _1$	$\eta _2$		$\tau$	$\eta _1$	$\eta _2$	
$\psi$	1.98															
$\tau$	.05	0.11			0.04				0.10				0.15			
$\eta _1$	.24	$-$ .14	1.00		$-$ .03	1.00			$-$ .17	1.00			$-$ .07	1.00		
$\eta _2$	.17	$-$ .06	.43	1.00	$-$ .04	.43	1.00		$-$ .09	.43	1.00		$-$ .04	.43	1.00	
C/IER category probabilities																
	$\kappa _0$	$\kappa _1$	$\kappa _2$	$\kappa _3$	$\kappa _0$	$\kappa _1$	$\kappa _2$	$\kappa _3$	$\kappa _0$	$\kappa _1$	$\kappa _2$	$\kappa _3$	$\kappa _0$	$\kappa _1$	$\kappa _2$	$\kappa _3$
	.14	.34	.41	.11	.15	.46	.31	.07	.10	.40	.41	.09	.09	.39	.44	.08

*Notes:* RT: item-level response times reconstructed from raw log events; TT: total time spent on screen divided by the number of items $J_s$; AAT: average item-level response time; TTFRT: difference between total time spent on screen and time to the first response divided by $J_s-1; \psi$: attentiveness; $\tau$: speed; $\eta _1$: environmental awareness; $\eta _2$: enjoyment of science; $\beta _C$ and $\sigma ^2_C$ give the mean and variance of the inattentive response time distribution.

#### Comparison with indicator-based procedures

In total, the conservative and liberal multiple-hurdle approaches classified 9.91% and 22.83% respondents as careless, respectively (see Table 1). That is, the liberal threshold settings yielded the highest C/IER rate out of all approaches considered and by far exceeded even those obtained from the model-based procedure drawing on TTFRT. The C/IER rate under the conservative threshold settings were similar to those of the model-based procedure drawing on TT and AAT. Under the conservative threshold settings, the C/IER rate goes back to 64 respondents not passing the RT hurdle, 147 respondents failing to pass the long string hurdle, and 135 respondents not passing the Mahalanobis distance hurdle. Under the liberal threshold settings, 401, 46, and 244 respondents did not pass the RT, long string, and Mahalanobis distance hurdle, respectively.

**Fig. 3:**
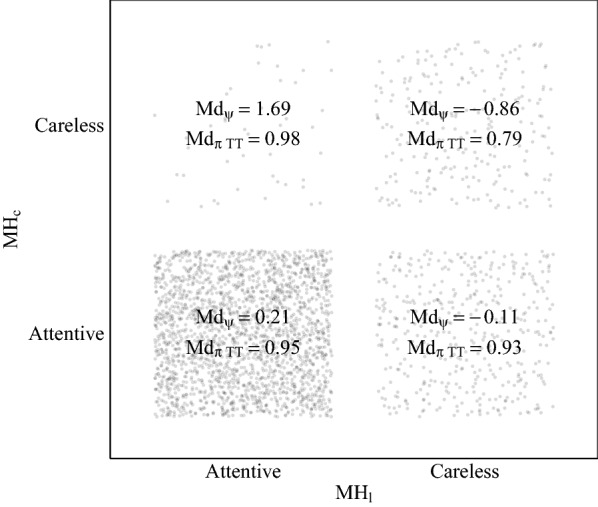
Agreement between the different approaches. Each dot represents a respondent. MH$_{\text {c}}$ and MH$_{\text {l}}$ denote the multiple-hurdle approach with conservative and liberal threshold settings, respectively; Md$_\psi$: median attentiveness parameters from the model-based approach using item-level RTs; Md$_{\pi TT}$: median attentiveness probabilities from the model-based approach using total time spent on screen

Figure 3 investigates agreement in the classification of respondents under the conservative and liberal threshold settings. The two threshold settings agreed in classifying respondents as attentive and careless in 2153 and 238 cases, respectively. The liberal threshold settings marked 412 respondents as careless that were classified as attentive under the conservative threshold settings, while the opposite was true for only 44 respondents. That is, employing more liberal thresholds lead to adding new respondents to the group of careless respondents rather than identifying different respondents as careless. To investigate agreement between the multiple-hurdle and model-based approaches, Fig. 3 displays median attentiveness parameters from the model-based approaches using item-level RTs and TT, yielding the lowest and highest C/IER rates out of all model-based approaches, for each of these four groups of respondents. Despite differences in overall C/IER rates, we observed agreement between the model-based and multiple-hurdle approaches in that respondents identified under both threshold settings as careless had markedly lower median attentiveness parameters than those identified as attentive under both threshold settings. Median attentiveness parameters in the group of respondents classified as careless under the liberal but attentive under the conservative threshold settings were between those obtained for the two groups where the different threshold settings agreed in their classifications. Interestingly, respondents classified as careless under the conservative but attentive under the liberal threshold settings yielded the highest median attentiveness parameters. Due to the small group size, however, this result needs to be interpreted with caution.

## Discussion

We presented a model-based approach that leverages response time (RT) information for identifying careless and insufficient effort responding (C/IER). This was achieved by integrating theoretical considerations on C/IER in non-cognitive assessments with recent model developments for identifying non-effortful behavior in cognitive assessment data (Ulitzsch et al., 2020). In doing so, the presented approach overcomes major limitations of previous methods for detecting and dealing with C/IER.

First, as a purely model-based approach, the presented approach does not require setting cut-off values for classifying C/IER. Rather, C/IER is identified employing mixture modeling techniques, assuming different data-generating processes for responses and RTs associated with C/IER and attentive behavior. Second, the approach can detect and deal with multiple response patterns arising from C/IER. This is done in a single step and does not require making assumptions on specific C/IER patterns that may be present in the data at hand. Third, by employing a latent response approach with attentiveness probabilities modeled as a function of person and item parameters, the approach allows for C/IER behavior to vary on the screen-by-respondent level as well as to assess screen and respondent characteristics associated with such behavior. Allowing for C/IER behavior to vary across the computer-administered questionnaire also allows keeping information from attentive responses of respondents who showed C/IER on some but not all screens. Further, the employed latent response approach supports considering differences in attentiveness when estimating the traits to be measured.

The approach comprises model classes for both item- and screen-level timing data. Item-level RTs potentially allow for a more precise depiction of response processes, however, are oftentimes not readily available. Conversely, screen-level timing data can easily be recorded with common tools for computer-administered questionnaires, making the approach readily applicable for typical data sets and do not require the collection of raw log events. The model for screen-level timing data poses a simplified version that—similar to previous approaches for C/IER—allows to identify C/IER at the respondent level. As such, it gives up some of the advantages of employing a latent response approach. Investigating parameter recovery, we found the model drawing on item-level RTs to yield unbiased estimates even under conditions with sparse information on C/IER behavior. Simplifying the model and using aggregated RTs led to overestimating the extent of C/IER behavior. Nevertheless, estimates of the correlations between traits were still unbiased under the investigated conditions. Correlations between traits commonly pose parameters of interest for applied researchers. Hence, in the case that no item-level RT information is available, the model for aggregated RTs can be used for screening for C/IER behavior and retrieving valid conclusions concerning the traits to be measured. When doing so, researchers should keep in mind that proportions of C/IER may be biased. We further note that, based on results from the empirical example, we simulated attentiveness to be only weakly related to the traits to be measured. When this is not the case and C/IER prevalences are higher, falsely assuming attentiveness to be unrelated to the traits to be measured, as done in both the model for aggregated RTs as well as customary indicator-based procedures, may yield biased conclusions (see Ulitzsch et al., 2020).

The approach was illustrated on data from the German PISA 2015 background questionnaire, employing different RT measures. We could show that the presented approach yields meaningful results for all RT measures and that the models employing different RT measures did not impact conclusions on trait correlations. Differences in conclusions concerning the prevalence of C/IER behavior corroborated those observed in the simulation study. Further, different RT aggregates that differed in whether they contained reading time yielded different conclusions on C/IER prevalence. We further illustrated the advantages of the proposed approach over previous indicator-based procedures. To this end, we showed how conclusions drawn on the basis of indicator-based procedures are heavily dependent on threshold settings, with vast differences being observable even for small differences in the exemplarily employed thresholds. Note that the difference between the different threshold settings was much larger than those between different RT aggregates, suggesting that decisions on thresholds are much more critical than decisions on the RT aggregate employed when no item-level RTs are available.

### Limitations and Future Research

The proposed approach’s component models for attentive and C/IE responses and timing data are formulated based on theoretical considerations on response behavior. Further, agreement with previously validated indicator-based procedures in that respondents with lower attentiveness parameters were at greater risk of being identified as careless in multiple-hurdle approaches provided first supporting validity evidence for the proposed approach. Nevertheless, further research on the approach’s validity for identifying C/IER is needed. Here, both studies conducted in the context of non-cognitive assessments (e.g., Meade & Craig, 2012; Niessen et al., 2016) as well as in the context of cognitive assessments may serve as blueprints (see Ulitzsch, Penk, von Davier, & Pohl, 2021, for a validation of the SA+E model for rapid guessing behavior)

Further, investigating to which extent reading time carries valid information on C/IER behavior is a pertinent topic for future research. This is a question that can only be addressed by a combination of theory and empirical research. In the case that C/IE respondents can be assumed to evaluate the question stem in a manner comparable to attentive respondents, reading time would pose a nuisance that confounds speed with which respondents read the question stem with differences in attentiveness and therefore should be left out when identifying C/IER on the basis of RT data. Conversely, in the case that respondents are assumed to skip reading the question stem, reading times should be considerably shorter and would, as such, pose a valid source of additional information on inattentiveness. Combinations of the two mechanisms may also be present in empirical data. Results from studies investigating these issues could then be integrated with the presented approach for an even finer-grained depiction of response behavior.

It should also be noted that the FSM used to reconstruct item-level RTs rests on assumptions on how respondents evaluate and respond to items that may be violated in practice. Examples for violations may be respondents that do not start by reading the question stem (Kroehne et al., April 2019) or respondents that first cognitively evaluate all items and then “bundle” the technical processes of choosing their answers, resulting in long times until their first response and only short times elapsing between subsequent responses. If that is the case, aggregated RT information that does not rely on these assumptions might pose a more stable and reliable source of information on respondents’ answering behavior. This issue could be addressed by simulating data that differ in whether or not assumptions of the FSM used to reconstruct item-level RTs from raw log events hold. The performance of different RT measures could then be compared to identify conditions under which each measure gives the most accurate estimate of the prevalence of C/IER behavior.

Regardless of the specific type of RT information employed, the presented approach heavily relies on this information for identifying C/IER. Hence, violations of assumptions on data-generating processes underlying RTs associated with attentive and C/IE responses may potentially result in misclassifications (see Molenaar, Bolsinova, & Vermunt, 2018). For instance, when the distance–difficulty effect as incorporated in the component model for attentive RTs does not adequately capture the relationship between attentive RTs and trait levels, assumptions on data-generating processes underlying attentive RTs are violated. To address this, a better understanding of the cognitive processes underlying attentive RTs in questionnaire data is urgently needed. A further possible violation of assumptions are changes in speed due to, for instance, warming up effects (Weitensfelder, 2017). By allowing for item-specific time intensity offsets, the model for item-level RTs can capture changes in speed that are shared by all respondents. The model cannot deal, however, with changes in speed that vary across respondents. To accommodate this, the presented approach may be extended by a growth curve model for speed (Fox & Marianti, 2016). To make mixture modeling approaches more robust to violations of distributional assumptions, Molenaar et al. (2018) suggested to employ a semi-parametric approach by categorizing RTs that could also be integrated with the presented approach.

As it is the case with previous behavioral measures of C/IER in non-cognitive assessment data (Huang et al., 2012; Meade & Craig, 2012; Niessen et al., 2016) and (rapid) guessing in cognitive assessment data (Nagy & Ulitzsch, 2021; Ulitzsch et al., 2020; Wang & Xu, 2015; Wise, 2017), the presented approach assumes inattentiveness to manifest itself in responses that do not reflect the construct to be measured. It does not consider C/IER that reflects the construct to be measured to some degree, which may occur when respondents skip lengthy instructions (Maniaci & Rogge, 2014) or read the item but do not put effort in retrieving the relevant information (see Ulitzsch et al., 2021, for a discussion of non-effortful responding in cognitive assessment). Further, the empirical application indicated that the model can not deal well with outrageously long RTs. These may stem from both attentive and C/IE response processes. Long attentive RTs may stem from respondents having problems understanding the questions or being indecisive between different response options. In online-administered questionnaires, long inattentive RTs may stem, for instance, from switching to another browser tab and subsequently providing a C/IE response. Using the proposed approach, we can already model very short RTs, assuming that these are likely to stem from C/IER behavior. Better understanding other types of C/IER behavior as well as the behavior underlying the occurrence of very long RTs and subsequently integrating these behaviors with the proposed approach remains an important task for future research.

A strength of the approach is that it can detect and deal with various types of C/IER at once. The price for this is that it does not allow for inferences on the specific types of C/IER. We can identify respondents with C/IER behavior, but do not know which type of C/IER behavior is shown. Note, however, that researchers are usually only interested in unbiased estimation of trait levels (i.e., accounting fo C/IER behavior), but not necessarily in the specifics of C/IER behavior. In case these are of interest, one may investigate response patterns of respondents with low attentiveness estimates and scan for specific patterns (e.g., straight or diagonal lining). If the goal is to model specific types of response styles, other approaches might be more appropriate.

It should also be noted that the scalability of the presented approach to the analysis of data with large samples and a high number of investigated constructs is limited, as, due to model complexity, infeasible running times may be encountered. Although we expect this issue to resolve with algorithmic and computational advances, for now, under such data constellations, researchers may find heuristic indicator-based approaches to be more practical for gauging the extent of C/IER in the data at hand.

The proposed approach allows for identifying and modeling C/IER in data retrieved from computer-administered questionnaires and, thereby, increasing the validity of inference drawn from such data. Implementing the proposed approach for real-time estimation of attentiveness poses a highly promising extension. Doing so would allow to monitor C/IER during the assessment procedure, issue warnings once pre-defined thresholds of acceptable aberrances are exceeded, and nudge respondents to provide more valid responses, thus increasing data quality. In experimental settings, Huang et al. (2012) as well as Wise, Bhola, and Yang (2006) already demonstrated the positive effects of warnings on attentive responding in both cognitive and non-cognitive assessments.

The approach is aimed at improving the validity of conclusions drawn on C/IER prevalences in the data at hand as well as relationships among constructs of interest by identifying and modeling C/IE responses. When doing so, the approach does not only provide parameter values for the employed IRT model that are based on attentive responses only, but also provides information on the attentiveness of respondents. Future research may investigate what further information the attentiveness variable provides on respondents, e.g., whether it provides a behavioral measure of respondent’s personality (see Pohl, Ulitzsch, & von Davier, 2021, for a neighboring discussion on behavioral aspects impacting test results). In support of this, Bowling et al. (2016) could show that individual differences in C/IER behavior as reflected in response–pattern-based indicators are consistent across time and study situations, and that C/IER is related to acquaintance-reported personality as well as to college grade point average and class absences. The proposed approach provides a sophisticated tool for furthering investigations of the additional information contained in response behavior and its relevance for real-life outcomes.

Note that, although not as widely available as RTs from cognitive assessments, in the context of non-cognitive assessments, item-level RT data become increasingly available (see Henninger & Plieninger, 2020, for recent studies; and Tunguz, November 2018, for a publicly available large-scale personality inventory data set with item-level RTs) or can be reconstructed using FSMs (Kroehne, 2019; Kroehne & Goldhammer, 2018). The present study showcased the utility of item-level RTs to gain a finer-grained understanding of respondents’ behavior in general and identifying C/IER behavior in particular and can as such be understood as a call for recording this rich source of information in non-cognitive assessments.

### Supplementary Information

Below is the link to the electronic supplementary material.

Supplementary material 1 (pdf 97 KB)

## Figures and Tables

**Fig. 4:**
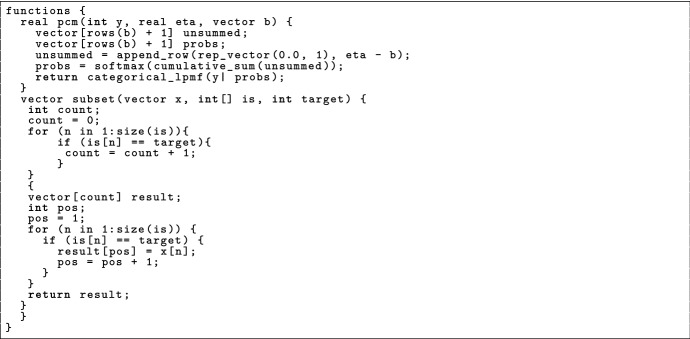
Function for **a** the partial credit model as well as **b** for subsetting vectors, used for identifying middle step difficulties

**Fig. 5:**
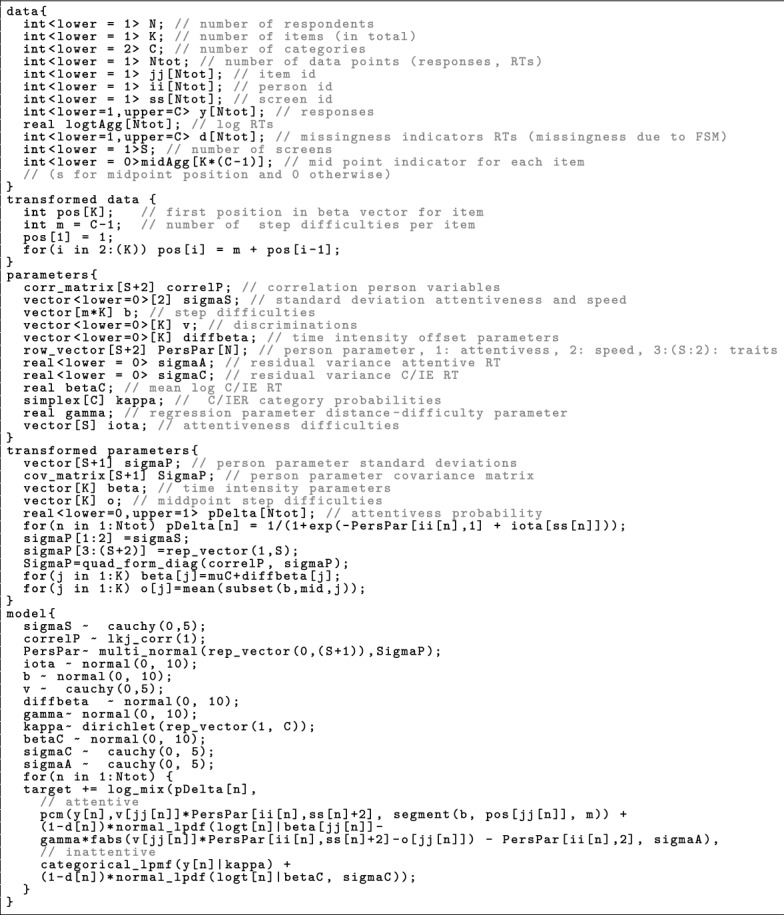
Stan code for the model with item-level RTs

**Fig. 6:**
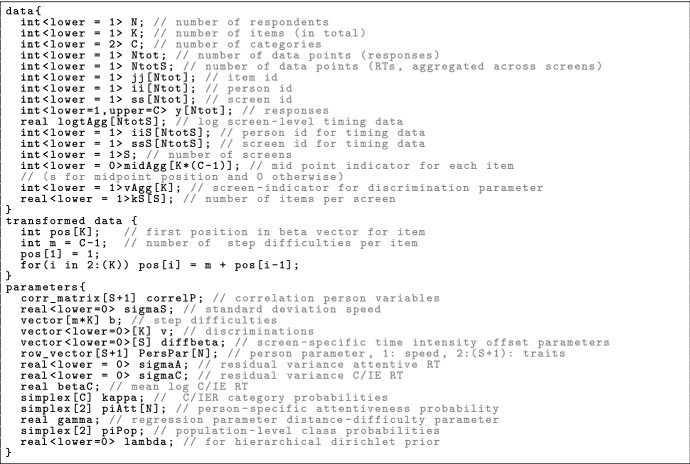
Stan code for the model with aggregated RTs (part I)

**Fig. 7:**
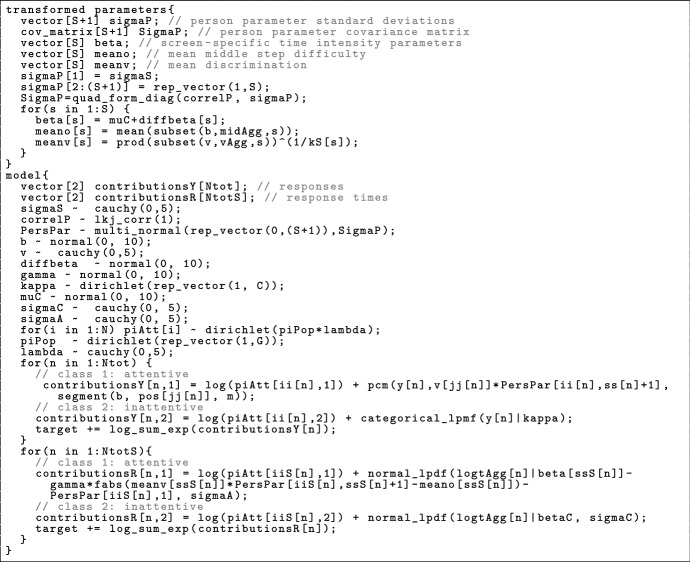
Stan code for the model with aggregated RTs (part II)

## Stan Code

See Figs. 4, 5, 6 and 7.

## Footnotes

1 Throughout this article, we employ the terms questionnaire, survey, and non-cognitive assessment data interchangeably.

2 Note that both filtering out too few or too many respondents can jeopardize validity of inferences. When too few respondents are filtered out, attentive responses are left confounded with C/IE responses. The effects of this are well studied (DeSimone et al., 2018; Huang et al., 2012; Schmitt & Stuits, 1985; Woods, 2006). When too many respondents are filtered out, valuable information contained in attentive responses is discarded. Further, the filtering procedure may systematically exclude specific subgroups of respondents, e.g., those with high trait levels when all items are worded in the same direction and the threshold for the long string index is set too low. This, too, is likely to impact conclusions on associations between the measured traits.

3 This assumption is reasonable in most computer-administered questionnaires where the number of items presented per screen is not very high. In the PISA 2015 background questionnaire for instance, a median of 4 items was presented per screen (range: 1 to 16 items; OECD, 2017).

4 For an uneven number of answer categories, Molenaar et al. (2015) suggested to taken the average of the two middle step difficulties.

5 Item omissions and missing RT information are ignored in the presented model, that is, are assumed to be missing at random (MAR) given the observed data and parameters of the model. This is the current state-of-the-art procedure for dealing with omissions in survey data. Note that omissions tend to occur to a much lesser degree in non-cognitive as compared to cognitive assessments. In the data set considered for the empirical example, for instance, the omission rate was as low as 0.66%.

6 Note that due to the log transformations of item-level RTs and average RTs, $\beta _{s}$ does not correspond to the mean of the item-level time intensity parameters $\beta _{js}$.
