# Supplementary material for: A Response-Time-Based Latent Response Mixture Model for Identifying and Modeling Careless and Insufficient Effort Responding in Survey Data
Source: Psychometrika. 2021 Dec 2;87(2):593–619. doi: 10.1007/s11336-021-09817-7 (PMC9166878; doi:10.1007/s11336-021-09817-7)
Supplement: Supplementary file 1 — Supplementary material 1 (pdf 97 KB) [file 11336_2021_9817_MOESM1_ESM.pdf]

Supplementary Material

A Response-Time-Based Latent Response Mixture Model for Identifying and Modeling Careless and Insufficient Effort Responding in Survey Data

1 Simulation Study

The aim of the simulation study was threefold. First, we aimed at investigating parameter recovery of the model using item-level response times (RTs) under realistic conditions. Second, we aimed at showcasing that the proposed approach can indeed deal with different C/IER patterns. Third, we aimed at assessing the potential loss in accuracy resulting from model simplification and aggregating RT information.

1.1 Data Generation

We generated 100 data sets according to the model for item-level RTs. Data-generating values were chosen to resemble parameter estimates reported in the empirical example. For each data set, we considered a sample of $N = 500$ respondents being administered $S = 2$ scales, measuring 2 traits with four four-point Likert scale items each. Scales for each trait are assumed to be presented on one screen each. To achieve a C/IER rate of 5%, we set screen attentiveness difficulties to $\iota_1 = -4.00$ and $\iota_2 = -3.00$. Attentiveness, speed, and trait variances were set to $\sigma^2_\psi = 1.50$, $\sigma^2_\tau = 0.10$, and $\sigma^2_{\eta_s} = 1.00$. Correlations of attentiveness with speed and each trait were set to $cor(\psi, \tau) = .10$ and $cor(\psi, \eta_s) = .20$. Correlations between speed and each trait were set to $cor(\tau, \eta_s) = -.10$. Correlations between traits were set to $cor(\eta_s, \eta_{s'}) = .40$. For each scale, middle item step difficulties were set to $b_{1s2} = -1.00$, $b_{2s2} = b_{3s2} = 0.00$, and $b_{4s2} = 1.00$. Lower and upper step difficulties were set to be symmetric with ± 1.00 around the middle step difficulty. Discriminations were set to $\nu_{1s} = 0.75$, $\nu_{2s} = \nu_{3s} = 1$, and $\nu_{4s} = 1.25$. Time intensity offset parameters were set to $\beta^*_{1s} = 0.25$, $\beta^*_{2s} = \beta^*_{3s} = 0.50$, and $\beta^*_{4s} = 0.75$.[1] The common mean of log inattentive RTs was set to $\beta_C = 0.65$. Variances of attentive and C/IE RTs were set to $\sigma^2_A = 0.65$ and $\sigma^2_C = 1.75$. We set the distance-difficulty parameter to $\gamma = 0.05$.

Following Curran and Denison (2019), we considered a scenario with different C/IER patterns. Doing so allows illustrating that the proposed model indeed can deal with various patterns arising from C/IER, as long as C/IE responses do not reflect the trait to be measured

[1] In preliminary analyses, we also investigated the impact of time intensity offset parameters close to zero, resulting in almost the same expected values for log attentive and inattentive RTs. Due to the different structures imposed on attentive and inattentive RTs, with attentive RTs assumed to be governed by respondents' speed, the respective RT distributions could still be separated well and we observed parameter recovery comparable to the conditions presented here.

and, on average, are not slower than attentive responses. Respondents were randomly partitioned into four equally-sized groups, each representing a C/IER pattern. For the first group of uniform random responders, C/IER category probabilities were set to κ_1 =(.25, .25, .25, .25). In the second group, representing random responding around the endpoints, respondents were randomly partitioned to subgroups with κ_{2a} =(.00, .15, .35, .50) and κ_{2b} =(.50, .35, .15, .00), corresponding to marginal category probabilities of κ_2 =(.25, .25, .25, .25). For generating straight lining behavior, the first C/IE response was chosen randomly with equal category probabilities and all subsequent responses were set to be the same as the first. This yielded marginal response categories of κ_3 =(.25, .25, .25, .25). Likewise, for simulating diagonal lining, for the fourth group, the first answer was determined randomly. Next, with equal probabilities, respondents were chosen to move upwards or downwards by one category on each item. If respondents reached either endpoint, subsequent responses were set to move away from the endpoint by one category on each item. Again, this yielded marginal response categories of κ_4 =(.25, .25, .25, .25). Hence, marginal probabilities for C/IE responses across all patterns are given by κ =(.25, .25, .25, .25).

1.2 Estimation Procedure

Each generated data set was analyzed with both the model for item-level and the model for aggregated RTs. We did not simulate reading time (i.e., time required for reading the question stem) and used the scale-level mean of item-level RTs as an aggregated RT measure. Stan code for both models is provided in the appendix of the article. All analyses were performed using R version 3.6.3 (R Development Core Team, 2017). Bayesian estimation was conducted using Stan version 2.19 (Carpenter et al., 2017) employing the rstan package version 2.19.3 (Guo, Gabry, & Goodrich, 2018). For all models, we ran four Markov chain Monte Carle (MCMC) chains with 4,000 iterations each, with the first half being employed as warm-up. The sampling procedure was assessed on the basis of potential scale reduction factor (PSRF) values, with PSRF values below 1.10 for all parameters being considered as satisfactory (Gelman & Rubin, 1992; Gelman & Shirley, 2011).

1.3 Results

For both models, we observed good quality of the sampling procedure and no replications with PSRF values below 1.10 were encountered. To evaluate bias and efficiency of parameter estimates, we assessed the median and 90% ranges of posterior means. Results for person parameter variances and correlations, the distance-difficulty parameter as well as proportions of attentive responses for the model with aggregated RTs are given in Figure 1.1. Person parameter variances and correlations as well as the distance-difficulty parameter were well recovered under both models. When screen-level timing data were employed, however, population-level attentiveness proportions were underestimated by approximately 4%, i.e., attentive responses were misclassified as C/IE responses.[2]

Figure 1.2 displays results for the item parameters of the measurement models for attentive and C/IE responses and RTs. Since we did not observe differences in parameter recovery for different data-generating values, we averaged results, displaying the median

[2]In preliminary analyses, we also investigated whether these misclassifications were less pronounced when items were equally targeted in terms of both step difficulties and time intensities as well as under conditions with equal C/IER rates across screens. These factors, however, did not improve estimation of attentiveness proportions.

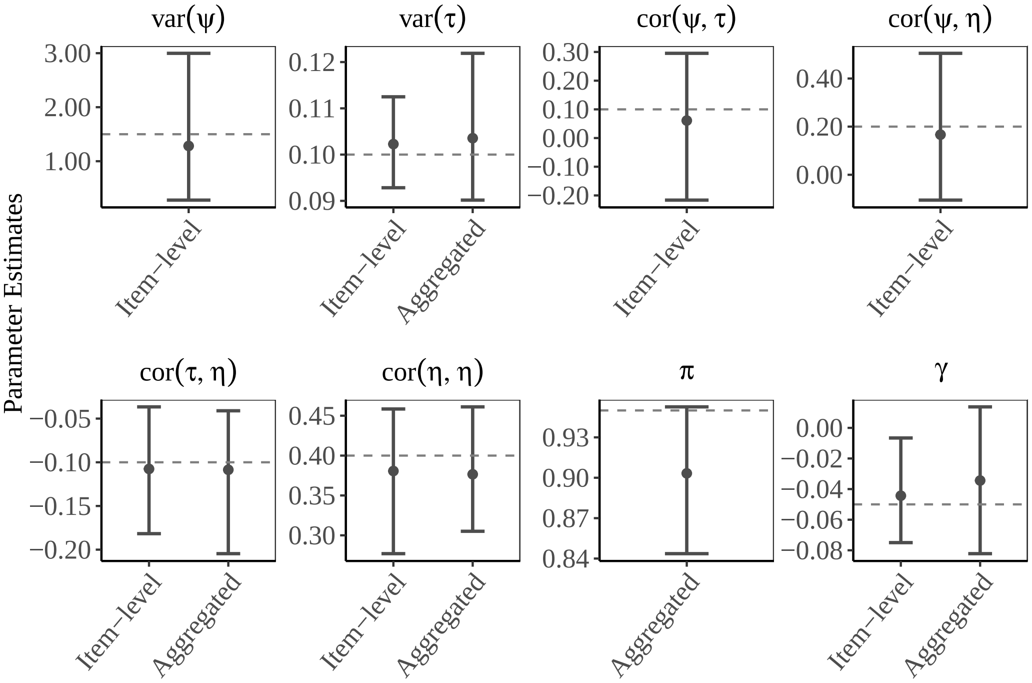

Figure 1.1. Medians and 90% ranges of person parameter variance and correlation estimates, the proportion of attentive respondents, and the distance-difficulty parameter. The dashed horizontal line indicates the respective true parameter. Note that y-axes differ in scale. ψ: attentiveness (only estimated in the model for item-level response times); τ: speed; η: trait; π: proportion of attentive respondents (only estimated in the model for aggregated response times); γ: distance-difficulty parameter.

and 90% ranges of posterior means against the mean of the different data-generating values for each parameter type. Using item-level RTs yielded good parameter recovery for parameters of the measurement models. Due to the rather small sample size of $N = 500$, discriminations were slightly upwardly biased (median of 1.05 as compared to the mean of data-generating parameters of 1). The misclassification of attentive as C/IE responses when using aggregated RTs was also mirrored in biased estimates of parameters related to the RT measurement model. Since data were simulated such that attentive RTs were in general higher than inattentive RTs, the misclassification was accompanied by underestimation of time intensity offset parameters for attentive RTs and overestimation of the common mean of inattentive RTs. Further, due to aggregating RTs, information on item-level variability in RTs was lost, resulting in estimates of residual variances σ_A^2 and σ_C^2 close to zero. Marginal category probabilities were recovered without bias when using aggregated RTs. They were, however, considerably more variable than estimates retrieved from the model with item-level RTs. The same was true for step difficulties. In addition, discriminations were markedly more upwardly biased when using aggregated RTs.

As evidenced in Figure 1.3, displaying median and 90% ranges of differences between attentiveness difficulty posterior means and data-generating values, attentiveness difficulties were well recovered in the model with item-level RTs. Although estimates were slightly more variable, this was even true for $\iota_s = -4.00$, corresponding to a scale-level C/IER rate of as low as 2%.

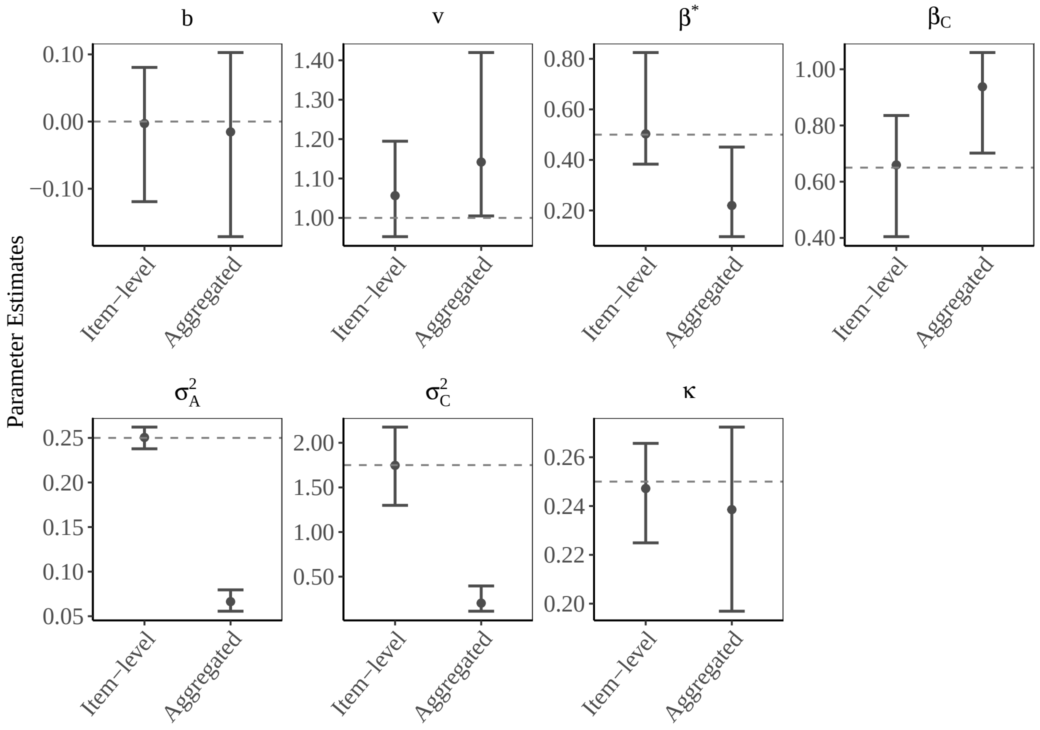

Figure 1.2. Medians and 90% ranges of parameters of the measurement models for attentive and careless and insufficient effort responses. The dashed horizontal line indicates the mean of the respective true parameters. Note that y-axes differ in scale. b: step difficulties; v: discriminations; β^*: time intensity offsets; β_C: mean of inattentive log response times; σ_A^2: residual variance of attentive log response times; σ_C^2: variance of inattentive log response times; κ: marginal careless and insufficient effort response category probabilities.

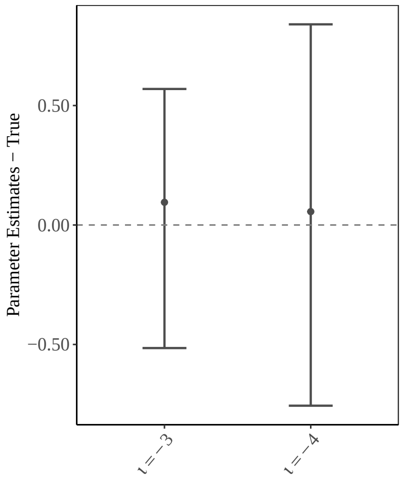

Figure 1.3. Medians and 90% ranges of differences between estimated and true attentiveness difficulties ι retrieved from the model for item-level response times plotted against the true parameters. The dashed horizontal line indicates a difference of zero.
